# Drivers of and Solutions for the Overuse of Antidepressant Medication in Pediatric Populations

**DOI:** 10.3389/fpsyt.2020.00017

**Published:** 2020-02-13

**Authors:** Lisa Cosgrove, Zenobia Morrill, Michelangela Yusif, Akansha Vaswani, Sadie Cathcart, Rebecca Troeger, Justin M. Karter

**Affiliations:** Department of Counseling and School Psychology, University of Massachusetts Boston, Boston, MA, United States

**Keywords:** pediatric depression, global mental health, psychiatry, antidepressant, overuse, misuse

## Abstract

Children in the United States and internationally are increasingly being diagnosed with depression and related psychiatric conditions and a recent study found that antidepressant (ADM) use in children and adolescents rose substantially in youth cohorts in five Western countries from 2005 to 2012. However, there has been ongoing controversy over the effectiveness and safety of ADM use in children, including concerns about ADM increasing suicidality and self-harm. In addition to the increase in the diagnosis of depression, commercially driven off-label prescriptions have been cited as a significant reason for high rates of pediatric ADM prescribing. In this commentary, we discuss two drivers of the overuse of ADM, both of which are products of an increasingly medicalized approach to mental health: 1) the demand for mental health and depression screening in youth, despite the lack of evidence to support it, and 2) the renewed momentum of the Global Mental Health Movement and concomitant calls to “scale up” the diagnosis and treatment of mental illness. Using the lens of institutional corruption, we identify the ways in which both guild and financial conflicts of interest create obstacles to rational prescribing practices in pediatric populations and offer suggestions for reform.

## Introduction

In his 2017 report to the United Nations (UN) on adolescent health, child psychiatrist and Special Rapporteur^1^ Dainius Pūras called upon states to develop “adolescent-friendly psychosocial interventions at the community level” (1) and emphasized the importance of avoiding “the excessive use of psychotropic medications” (p. 19). Two years later he reiterated his concerns about the far-reaching harms of over-medicalization to children, noting that “global trends to medicalize complex psychosocial and public health issues in childhood should be addressed with a stronger political will” (2019, p. 12). The Special Rapporteur's observations and recommendations are critically important and timely given the recent finding that antidepressant medication (ADM) use in children and adolescents rose substantially in five Western countries from 2005 to 2012 (2).

Indeed, youth in the United States and internationally are increasingly being diagnosed with depression and related psychiatric conditions (3). Rates of major depressive episodes increased 52% from 2005 to 2017 among adolescents aged 12 to 17 and 63% from 2009 to 2017 among young adults aged 18–25 (4). The reasons for this increase are multifaceted and impossible to determine, but Twenge et al. (4) posit the rise of electronic communication, digital media, and declines in sleep duration as potential explanations. In terms of biomedical interventions for depression, there has been ongoing controversy over the effectiveness and safety of ADM use in children (5), including concerns about ADM increasing suicidality and self-harm (6–8). However, it should be noted that there is more evidence to support use of ADM for anxiety (9, 10).

Certainly the increase in depression diagnoses has contributed to the increase in ADM prescribing in youth. In this commentary, we discuss two other drivers of the overuse of ADM: 1) the demand for mental health and depression screening in youth, despite the lack of evidence to support it, and 2) the renewed momentum of the Global Mental Health Movement and concomitant calls to “scale up” the diagnosis and treatment of mental illness. In contrast to the over-medicalization of distress that characterizes much of adolescent mental health care, we argue for a more conservative and contextual approach. Using the lens of institutional corruption (11), we identify the ways in which guild and financial conflicts of interest create obstacles to rational prescribing practices in pediatric populations and offer suggestions for reform.

### Depression Screening in Adolescents: Is There Evidence of Benefit?

“All screening programmes do harm; some do good as well” [(12), p. 480].

Screening has been enthusiastically embraced for almost half a century, beginning with the use of mammography to identify cases of breast cancer (13). Screening is premised on the idea that early identification of a pre-clinical disease in asymptomatic people leads to effective interventions that improve health outcomes. It is hoped that questionnaire-based depression screening will identify undetected cases of depression. Thus, some readers might ask, “How could screening for depression be harmful?” However, screening for depression is only useful when it improves outcomes beyond those of standard care (14–16). For example, a 2017 systematic review of the clinical trial evidence for depression screening among children and adolescents (17) found no direct randomized control trials (RCTs) evidence to support it. The researchers noted there would be unintended harm from screening and recommended that there should be careful consideration “of potential harms, as well as the use of scarce health resources, that would occur with the implementation of screening programs” [(17), p. 813].

Additionally, an examination of recommendations from three national guideline organizations (17) revealed a lack of evidence to support questionnaire-based screening. The recommendations for screening for alcohol misuse, depression, developmental or speech and language delays, domestic violence, and suicide risk from the United States Preventive Services Taskforce (USPSTF), the Canadian Task Force on Preventative Health Care (CTFPHC), and the United Kingdom National Screening Committee (UKNSC) were reviewed. Only six RCTs that assessed the benefits of screening over standard care were cited across all of the recommendations made for or against questionnaire-based screening. A closer inspection of the six RCTs cited revealed that in five of the trials, no statistically significant primary or secondary health outcomes from screening were found. Confidence in the one trial that reported equivocal results was compromised by the misreporting of outcomes (18). The CTFPHC and UKNSC have made 11 recommendations against the use of questionnaire-based screening, including against routine screening for depression. In contrast, the USPSTF recommended questionnaire-based screening for depression and three other conditions and made no recommendations against screening.

The lack of evidence for screening may initially seem surprising, in part because the terms “screening” and “assessment” are often conflated. However, they are very different procedures. Screening for depression involves the use of brief questionnaires [typically the Patient Health Questionnaire or PHQ–9^2^; (19)] to identify an already existing problem. A clinical assessment, on the other hand, refers to a thoughtful, contextual, and individualized conversation between a patient and healthcare provider.^3^ Thus, it should be emphasized that recognizing the lack of evidence to support questionnaire-based depression screening is not the same as advocating for a “don't ask don't tell” policy and practice. Individualized clinical assessments may also contribute to more accurate estimates of national depression prevalence and help reduce overdiagnosis and overtreatment.

There are many reasons why applying a questionnaire-based screening model to presently experienced problems, such as depression, may not be effective and may result in more harm than benefit. First, depression is not analogous to infectious diseases such as HIV or hepatitis C. Research has shown that depression, particularly more mild forms, and, notably, in the case of children and adolescents, can resolve without intervention (20–22). Depression is not necessarily progressive, and certainly not progressive in the same way as an infectious disease. Additionally, its status as a disease codified in the Diagnostic and Statistical Manual of Mental Disorders (*DSM*) has been challenged (23). Transient and contextual reasons can be the basis for an adolescent scoring “positive” on a depression screening instrument and substantial improvement in mood can result when these stressors abate (24). Second, there is the possibility of the nocebo effect, the negative or iatrogenic effect of placing labels on peoples’ distress, when they themselves have not categorized their experience nor sought a mental health diagnosis from a health care or school setting [see, e.g., (15, 25)]. Also, for screening to improve health outcomes there must be high quality evidence (preferably from randomized clinical trials) demonstrating its effectiveness and safety. In the case of antidepressant medication, there is substantial controversy regarding the risk-benefit ratio (26–30).

Despite the lack of evidence to support it, in 2016, the USPSTF reaffirmed their 2009 recommendation for screening adolescents age 12 and over (31, 32). In the 2016 update, the identification of specific ADMs and specific therapeutic interventions as well as the following cautionary statement were removed: “However, because of risk of suicidality SSRIs should be considered only if clinical monitoring is possible” [US Preventive Services Task Force, 2009 (33), p. 1224]. The stated rationale for removing the identification of specific agents and removing the concern about risk of suicidality with the use of SSRIs in adolescents, was the “recognition of *decreased concern over the harms of pharmacotherapy in adolescents*…” (emphasis added, p. 7). Pharmacotherapy is listed as the first treatment option (p. 7) and the Task Force concluded that “the evidence on the frequency of medication-related adverse events in adolescents is adequate to estimate that the magnitude of harms of pharmacotherapy is small if patients are closely monitored” (p. 6). This conclusion not only stands in contrast to the research which provides evidence of a questionable risk-benefit ratio for ADM use in youth (10, 34), but it may also lead to an increase in the use of ADM in pediatric populations because of the statement regarding “decreased concerns” about the safety of ADM.

As can be seen in this brief review, there is great enthusiasm for depression screening despite the lack of evidence of benefit over care as usual. To our knowledge, no methodologically rigorous studies have been conducted to indicate a reduction in depressive symptoms among students identified as at-risk through in-school screening procedures. The studies in primary care settings that do show modest benefit have been found to have significant design and reporting flaws (35). It is difficult to accept evidence that disrupts our firmly held beliefs and therefore it is understandable that both health care and school professionals continue to hope that screening will lead to better health outcomes for youth. However, when health care policy (e.g., USTSPF recommendation for screening youth) starts to look too much like an advertisement for cherished beliefs, we cannot claim to be engaging in evidence-based practice [see, e.g., (36)]. Moreover, routine depression screening may have the unintended effect of overtreatment with antidepressants and could deflect limited healthcare resources away from those who need it most (2, 18, 31, 37–39). Research demonstrates that a stepped approach is best: treating mild-moderate depression with an antidepressant has been found to be no better than watchful waiting (40, 41).

### Mind the Gap: Renewed Global Emphasis on Scaling Up the Diagnosis and Treatment of Depression

The Global Mental Health Movement (GMHM), an initiative aimed at scaling up the diagnosis and treatment of mental health disorders (particularly in low-middle income countries) may also result in the overuse of ADM in pediatric populations. In 2008, the World Health Organization (WHO) developed its Mental Health Gap Action Programme (mhGAP)^4^. The mhGAP identified depression as a priority disease category and was promoted as a resource for all countries, especially low- and middle-income. As part of this program, various guides were developed to increase the diagnosis and treatment of mental disorders. The mhGAP intervention guide was launched in an attempt to provide specific guidance for scaling up interventions—it functions as a type of “how to” manual for the management of mental disorders (42, 43). In October of 2018 the most recent *Lancet* Commission's Report was published, prioritizing child and adolescent mental health and advocating for early screening and intervention. Similarly, the WHO’s “Let’s Talk” 2017 campaign on depression identified adolescents and young adults as one of three populations disproportionately affected by depression.

These recent GMH initiatives focus on early detection. Calls to dramatically scale up depression care are made by appealing to economics and parity: The WHO estimates that there will be a loss of a trillion dollars, every year, between 2016 and 2030 because of the lost productivity and economic burden of mental illness (44, 45). The argument is made that increasing access to treatment and funding child and youth mental health screening is a sound monetary investment, especially in low and middle income countries—it will mitigate future unemployment and reduce welfare expenditures and criminality. In fact, the WHO predicts that every dollar invested in mental health will yield a USD 4-dollar gain (45).

The parity argument—mental health is no different from physical health—is also used to bolster claims that childhood disorders will inevitably progress into adult mental disorders. For example, the 2018 *Lancet* report strongly recommends identifying sub-threshold or sub-syndromal detection of mental disorders in order to intervene “before substantial disability sets in” [(46), p. 1564]. Although it was briefly noted that there are no diagnostic tests or tools that can accurately detect who will go on to develop a disorder or respond to an intervention, early detection is presented as unequivocally beneficial. In this way, the report reinforces the belief that there is an equivalence between mental and physical disorders in terms of both etiology and disease progression. This assumed equivalence, emphasis on halting disease progression, and the fact that the mhGAP program is based on a model that endorses biomedical psychiatry (47), positions Western psychiatric treatment as the main solution to the global mental health care crisis. In turn, this can lead to overzealous ADM prescribing, particularly in children and adolescents as they are identified within the GMHM as a population at risk.

It is also noteworthy that the movement to globalize mental health was formed across political and economic organizations (i.e., WHO, World Bank, International Monetary Fund), resulting in an uneasy alliance among psychiatry, public health, and international development (48, 49). There is increasing concern that the data reinvigorating the GMHM (e.g., disease burden estimates of depression) have been distorted by commercial interests and psychiatry's capture of this movement (50). The conceptual and normative framework of institutional corruption can illuminate the ways in which guild and commercial interests can lead to the over-diagnosis and over-treatment of youth internationally (i.e., through GMHM initiatives and programs). That is, institutional corruption, unlike individual corruption, is not about unethical people behaving in morally bankrupt ways. Institutional corruption can be defined as a dependence that results in widespread or systematic practices that undermine the integrity of that institution or weaken public trust. Perhaps most important, the actors within the institution do not perceive themselves as being influenced by guild or financial interests or implicit bias. Unwittingly, organized psychiatry developed financial incentives (e.g., allowing leaders within the field to become the pharmaceutical industry's “key opinion leaders”), and behavioral norms (e.g., not requiring disclosure of financial conflicts of interest in previous editions of the *DSM*)^5^, that created an improper dependence on industry. It is improper in the sense that the processes for generating knowledge about the etiology and treatment of mental illness became compromised. Practices that allowed for a deviation from organized psychiatry's public health mission—and that also led to a distortion of scientific truths—became normalized (53).

Looking at organized psychiatry and the GMHM through this conceptual lens helps us see the economies of influence (e.g., individual and organizational ties to industry; guild interests) at play and their implications. Because (Western) psychiatry has dominated the GMHM, the implications are profound for public health. It is not surprising that proponents of the movement—who are mainly psychiatrists and psychologists—strongly advocate for scaling up diagnosis and pharmacological and psychotherapy interventions. Certainly, many youth in the U.S. and internationally are underserved and in need of treatment. However, the fact that the pharmaceutical industry and the mental health professions are obvious beneficiaries of scaling up efforts warrants more serious attention [see e.g., (50)].

For example, both the 2011 and 2018 *Lancet* reports were developed mainly by psychiatrists who have a guild interest in advocating for pharmacological interventions. It has been consistently shown—across multiple areas in medicine—that clinical practice guidelines and treatment recommendations produced solely by medical specialty groups, especially those with industry ties, make recommendations that are consistent with their guild interests (54). As a result, their recommendations are less conservative than those produced by more multidisciplinary groups (55). Also, many of the epidemiological studies reporting on the so-called international mental health crisis (and the economic burden incurred by it) were funded by the pharmaceutical industry (56, 57) and one of the largest forums on the global burden of depression (58) was sponsored by Lundbeck. However, researchers without industry ties note that the GMHM estimates of depression are unreliable and likely exaggerated (59) conducted a robust and methodologically rigorous study in which they identified primary and secondary data sources used in the global burden of depression estimates and assessed these sources in terms of completeness and representativeness (e.g., were they drawn from a nationally representative population). The authors found significant study design flaws and poor compliance with the inclusion criteria for the depression estimates and concluded that the “uncritical application of these estimates to international healthcare policy-making could divert scarce resources from other public healthcare priorities” [(59), p. 25].

The economies of influence within the GMHM create the risk of over-treatment and increased (but not evidence-based) ADM prescribing in pediatric populations worldwide. The framework of institutional corruption can be used not only to assess the role and extent of pharmaceutical funding of GMH programs and forums, but also to critically assess the ways in which guild interests and implicit bias can influence the *Lancet* Commission Reports. Because the conceptual and normative framework of institutional corruption is solution rather than blame oriented, it draws attention to the systemic practices that need to be addressed and highlights the need for epistemological pluralism in depression care.

### Solutions for Reform

“Capabilities rework recovery not from within (where it remains hostage to a rhetoric of suffering), but from without (informed by an idiom of opportunity). Not healing but equality becomes the operant trope” [(60), p. 9].

Certainly, there are profound corollaries to children's sadness (e.g., acting out, drug use, school and relationship difficulties), whether one labels that sadness from within a medical model (major depressive disorder) or from a more descriptive perspective [e.g., unhappiness, see (61)]. But labels do matter and how we think about a problem determines what we do about it. Diagnosing youth with major depressive disorder affects their self-understandings, creates certain pathways for the future, and forecloses others (62). It also frequently leads to long-term prescribing and polypharmacy. Thus, mental health professionals must be willing to see the ways in which institutional thinking and practice, and guild interests may impede their ability to genuinely make room for models of care that fall outside the medical model (63). Many psychiatrists are already doing this by recognizing the limited efficacy of ADM, advocating for conceptual models that focus on the underlying reasons for childhood depression, and by recommending context-rich explanatory models (61, 64–66). Over a decade ago a leading researcher did not mince words about the discrepancy between expectations of the effectiveness of ADM and the scientific reality:

The widely held clinical view of “antidepressants” as highly effective and specific for the treatment of all types of depressive disorders is exaggerated. This sobering conclusion is supported by recent findings from the National Institute of Mental Health (NIMH)-sponsored Enhancement Program for Bipolar Disorder (STEP-BD) and Sequenced Treatment Alternatives to Relieve Depression (STAR*D) projects. Antidepressants have limited short-term efficacy in unipolar depressive disorders and less in acute bipolar depression; their long-term prophylactic effectiveness in recurrent unipolar major depression remains uncertain [(67), p. 957].

Unfortunately, however, psychiatrists and other prescribing providers have not heeded Ghaemi's (67) cautionary words, and conversations about the diagnosis and treatment of depression in pediatric populations too frequently becomes contentious and polarized (i.e., pro *vs.* anti-medication). This commentary has tried to avoid this kind of polarization by arguing for a more conservative and contextualized approach to depression care. We have identified two potential drivers of inappropriate ADM prescribing that may lead to unintended harm. In the following section we offer specific suggestions for avoiding over-medicalizing emotional distress and for enhancing rational ADM prescription practices in pediatric populations.

1. As much as clinicians, researchers, and policymakers may want to believe that questionnaire-based depression screening will lead to better mental health outcomes for youth, the current evidence does not lend support for this belief. Therefore, if we want to engage in evidence-based practice, we need to accept the data and develop policies and programs in accordance with the (independent) scientific literature—rather than making policy recommendations based on well-intentioned (but unfounded) beliefs. Advocating for thorough, individualized assessments rather than routine screening is in the public's best interest. An individualized and stepped approach will facilitate more rational ADM prescribing for children and adolescents insofar as medication would be considered only after psychosocial support and counseling have failed to achieve results.
2. Using a stepped approach to inform short-term ADM prescribing is congruent with a rights-based and social determinants framework. However, bringing this framework to fruition will require a multi-perspective and multidisciplinary approach. For example, there needs to be greater inclusion of people who have been in the mental health care system, medical anthropologists, and sociologists in the GMHM. A multidisciplinary approach with genuine stakeholder involvement can off-set guild interests, expand our current ways of thinking, and help to stop the uncritical exportation of Western conceptualizations of distress (60, 68, 69). Most importantly, engaging with other disciplines will help us see how structural risk can become understood and codified as psychological risk—and how psychology and psychiatry may be inadvertently contributing to this problem.
3. Stronger “political will” is needed to develop policy initiatives and clinical practice that are aligned with a rights-based and social determinants framework—one that focuses on the socio-political context (e.g., poverty, inequality, violence) of emotional distress (70). Simply “scaling up” the diagnosis and treatment of depression in youth (e.g., *via* routine depression screening) is short-sighted and not evidence-based. The UN Special Rapporteur is correct: we must *shift the focus to the conditions that promote well-being* for communities, families, and children. As child psychiatrist Sami Timimi (61) astutely noted, although “unhappiness among children seems to be rising. [simply] labelling it as depression and prescribing antidepressants is ineffective and possibly harmful. It is time to focus on the underlying reasons” (p. 1394). Until we address the root causes of distress—such as government-led austerity measures that have given rise to poverty, unemployment, and the creation of unlivable conditions—we will not be able to make progress on health promotion efforts (71).
4. Although it is common practice to try multiple ADMs when there is a lack of response (72–74), a recent independent study found a negative influence of the number of prior ADM trials on treatment outcome. The number of prior ADM trials was associated with a greater odds of depressive relapse as well as a shorter time to relapse (75). Yet, industry affiliated researchers, clinicians, and clinical practice guidelines continue to recommend long term or even indefinite psychotropic medication treatment [see e.g., (76, 77)]. Thus, ADM should only be considered when there have not already been multiple prior (appropriate) drug trials, the limitations (in terms of effectiveness and adverse effects) are thoroughly considered and discussed, and when the patient is open and interested in medication.

## Author Note

LC is a Clinical Psychologist and Professor at the University of Massachusetts Boston where she teaches courses on psychiatric diagnosis and psychopharmacology, and she is a former Research Fellow at the Edmond J. Safra Center for Ethics, Harvard University. Her research addresses the ethical and medical-legal issues that arise in organized psychiatry because of academic-industry relationships. LC has published widely on these topics and her research has been cited and discussed in major media outlets.

## Author Contributions

LC conceptualized the study, developed the first draft of the paper (excluding the section on Global Mental Health) and reviewed and contributed to all subsequent drafts. ZM conceptualized the section on Global Mental health, developed the first draft of this section, and made significant contributions to all drafts. MY contributed to the development of the section on screening, reviewed the paper for accuracy, and made significant contributions to all drafts. AV drafted parts of the conclusion, sections on screening, and made significant contributions to all drafts. SC developed the section on screening in schools and participated in making subsequent revisions to the paper. RT contributed substantially to paper revisions and compiling background research and references. JK contributed to the conception of the paper, compiled background research and contributed to subsequent revisions.

## Conflict of Interest

The authors declare that the research was conducted in the absence of any commercial or financial relationships that could be construed as a potential conflict of interest.

## Footnotes

1 For the right of everyone to the enjoyment of the highest attainable standard of physical and mental health.

2 The development of the PHQ was funded by Pfizer.

3 It is noteworthy that early screening tools (e.g., U.S. military psychological screening; diabetes) were not implemented on a standalone basis, but rather as a precursor to a more thorough clinical interaction/evaluation [see e.g., (13)].

4 https://www.who.int/mental_health/mhgap/en/.

5 It is noteworthy that the co-chair of the DSM IV, in a response to a 2009 study (51) that found over 90% of the psychiatrists who wrote the clinical practice guidelines for mood disorders and schizophrenia had ties to drug manufacturers, told a USA Today reporter, "There's this assumption that a tie with a company is evidence for bias. But these people can be objective" (52).
